# Two courses of deconstructed coronavirus please

**DOI:** 10.1371/journal.ppat.1009547

**Published:** 2021-04-29

**Authors:** Connor G. G. Bamford

**Affiliations:** Wellcome-Wolfson Institute for Experimental Medicine (WWIEM), School of Medicine, Dentistry and Biomedical Sciences, Queen’s University Belfast, Belfast, United Kingdom; Icahn School of Medicine at Mount Sinai, UNITED STATES

In a study published recently in *PLOS Pathogens* [1], Ju and colleagues report the development of a safer way to find antiviral drugs against Severe Acute Respiratory Syndrome Coronavirus 2 (SARS-CoV-2). Using molecular virology approaches, scientists led by Qiang Ding from Tsinghua University, Beijing, exploited the concept of “*trans*-complementation” to create a deconstructed virus-like system to work with SARS-CoV-2 outside of the restrictive environment of a Biosafety Level 3 (BSL3) lab. The researchers, from 6 institutes across China, showcase their reagent to identify 5 potent antivirals and study fundamental aspects of coronavirus biology. This paper symbolises a growing collaborative movement in science heightened during the pandemic, which should be actively encouraged as we emerge from its shadow.

Since late 2019, SARS-CoV-2 has spread around the world, causing (as of March 2021) at least 100 million infections, nearly 3 million deaths, and countless instances of long-term disease. Economic and societal disruption lie in its wake, at levels not seen for decades. Furthermore, we are still in its midst, and although vaccines look very promising, the future is far from certain. In the absence of highly effective immunisations, pharmaceutical interventions can help protect against severe disease and even death. Although researchers have committed their careers to unpicking coronavirus biology and developing targeted antivirals, such as Remdesivir, their clinical efficacy is limited in the real world when patients arrive to hospital already in advanced stages of disease.

There therefore remains a great need to identify new anti-SARS-CoV-2 drugs that will be able to alleviate the burden on global healthcare systems. This search for new treatments may be best catered for by a diverse and dedicated worldwide scientific effort. This effort would be able to use creative screening platforms and broad compound libraries of approved and non-approved drugs to maximise the potential to discovery potent and safe antivirals. However, this global search effort is impeded by restrictions placed on working with “fully infectious” SARS-CoV-2 in BSL3 labs, due to the nature of the pathogen. Given that SARS-CoV-2 is pathogenic, and its transmission is under exceptional suppression, work on SARS-CoV-2 is rightfully only used under BSL3 conditions at this time.

**Fig 1:**
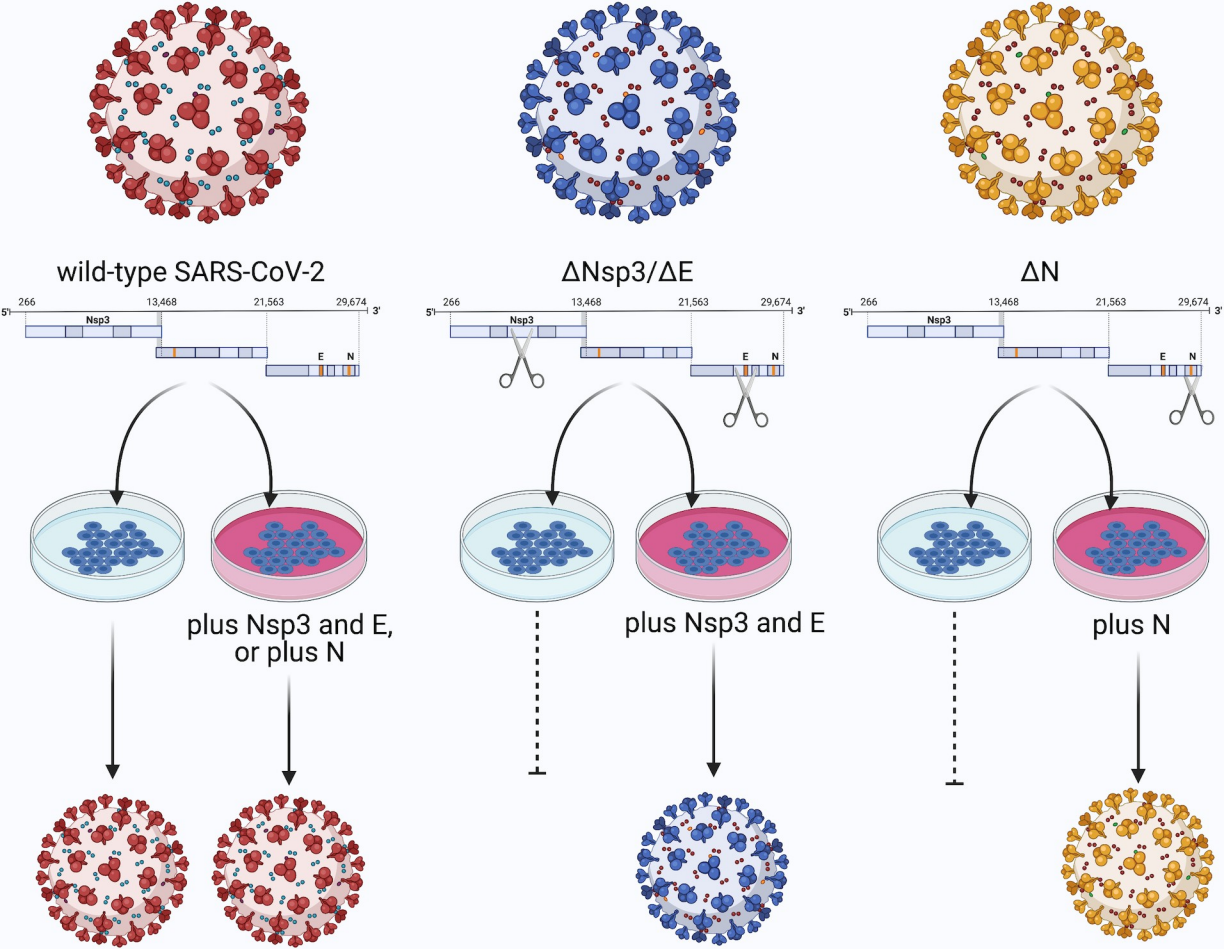
Generation of “biologically contained” single-cycle SARS-CoV-2 viruses using virus reverse genetics and “*trans*-complementation”. Wild-type virus and gene-deleted viruses are produced through construction and manipulation of full-length virus genomes. Wild-type virus can infect any susceptible cells, and outcoming virus is fully infectious. However, gene-deleted viruses (loss of Nsp3, E, or N) can only replicate in cells providing the essential deleted gene products “in *trans*.” Cells expressing the genes in *trans* will generally only be found within the lab, and not in somebody’s respiratory tract, making these systems much safer and capable to be used outside of a BSL3 lab. Created with BioRender.com. BSL3, Biosafety Level 3; SARS-CoV-2, Severe Acute Respiratory Syndrome Coronavirus 2.

The study of molecular virology over the last 50 years has allowed a greater control over virus biology in ways that can be exploited to help circumvent challenges such as those raised by SARS-CoV-2. It is this challenge that Xiaohui Ju and colleagues sought to overcome through the generation of a novel reagent, so-called trVLPs, or transcription and replication competent virus-like particles, to identify SARS-CoV-2 antivirals outside of BSL3 conditions (**Fig 1**). This system can be used to study the complete SARS-CoV-2 infection cycle from entry through replication to assembly. This tool was based on the knowledge that one coronavirus gene, N or nucleoprotein, is essential for replication as it positively regulates translation, replication, and assembly. Construction of a full-length SARS-CoV-2 DNA clone allowed the deletion of its N gene and replacement with a reporter gene green fluorescent protein (GFP) to measure infection. Provision of N “in *trans*” in a stable cell line rescues N-deficient virus. This provided N cannot be incorporated back into the genome and so infection is limited to a single round or replication, which is enough to find antiviral molecules.

As safety in working with SARS-CoV-2 is paramount, to reduce the potential even further for the N gene to recombine, Ju and colleagues utilised intein protein-splicing technology to effectively split N in 2 on independent genes. Although considered very unlikely, the only risk may be that while these modified viruses can still infect cells, coinfection in the respiratory tract of a worker carrying wild-type virus could provide opportunity for recombination and rescue of infectivity or that given the immense selection pressure, generation of N-like genetic novelty in vitro (as has been shown in E-deleted coronaviruses; [2]) could facilitate rescued multicycle infection.

Through the construction of their system, the Beijing-led team showed off their reagent to identify several effective antiviral drugs in vitro. Additionally, through reconstitution with distinct N genes, the group delineated the role of specific N features, such as the incompatibility between SARS-CoV-2 and N from the related Middle East Respiratory Syndrome Coronavirus (MERS-CoV), the critical role of specific N amino acid positions in promoting infection, and protein–protein interactions between N and host cell factors, rediscovering some previous identified interactions in other coronaviruses of animals [3].

*Trans*-complementation is a powerful technique in molecular virology to study basic biology and genetics. The system described in *PLOS Pathogens* here is not the only available system for SARS-CoV-2. Recently, Zhang and colleagues, from the lab of Pei-Yong Shi at the University of Texas Medical Branch, describe a similar reagent [4]. Unlike Ju and colleagues, the United States team removed 2 genes: “ORF3” and “E,” which are then provided in *trans*. Zhang and colleagues looked closely at the safety profile, showing that the single-cycle viruses created were apathogenic in rodents. In the past, similar systems have been published for other coronaviruses too by the lab of Luis Enjuanes for MERS-CoV [5] and the porcine transmissible gastroenteritis coronavirus (TGEV) [6]. Furthermore, while a fantastic tool for discovery science, single-cycle viruses like these can be used as a safer vaccination tool such as has been used for Ebola [7].

Endeavours like those pursued by Ju and colleagues highlight the need and potential for the democratisation of science in the face of significant societal challenges, if reagents and protocols are indeed adequately shared. There are numerous examples of this in the scientific community, such as the generation and sharing of SARS-CoV-2 pseudoparticles from the lab of Benhur Lee [8] and the construction of a Coronavirus Disease 2019 (COVID-19) toolbox from the MRC-University of Glasgow Centre for Virus Research [9]. It is hoped that efforts like this will enhance the efficiency of science, while not jeopardising the safety of researchers and their communities. While enhancing sharing may make an impact during the COVID-19 pandemic, it is likely to have greater impact in the future, such as the “next big one” or slower crises like antimicrobial resistance. Every effort should be taken to encourage this behaviour for our own benefit.
